# Supplementary material for: Positive cardiac inotrope omecamtiv mecarbil activates muscle despite suppressing the myosin working stroke
Source: Nat Commun. 2018 Sep 21;9:3838. doi: 10.1038/s41467-018-06193-2 (PMC6155018; doi:10.1038/s41467-018-06193-2)
Supplement: Supplementary file 1 — Supplementary Information [file 41467_2018_6193_MOESM1_ESM.pdf]

Positive Cardiac Inotrope Omecamtiv Mecarbil Activates Muscle Despite Suppressing the Myosin Working Stroke

Woody et. al

Supplementary Information

Supplementary Figures

Supplementary Figure 1 Ensemble Averages of Myosin Working Stroke at 200 nM MgATP 2

Supplementary Figure 2 Stroke Size Distributions at Saturating [ATP] 3

Supplementary Figure 3 Proportions of Events with 5.5 nm Stroke and Rate k_a as a Function of [OM] 4

Supplementary Figure 4 Distributions and Fits of the Kinetics of Detachment at Saturating [ATP] 5

Supplementary Figure 5 Distributions and Fits of the Kinetics of Detachment at Sub-saturating [ATP] 6

Supplementary Figure 6 Dissociation of Myosin from Actin from Nucleotide-free or ADP-bound States 7

Supplementary Figure 7 Isometric Force as a Fraction of Myosin Bound to OM 8

Supplementary Figure 8 Motility Rate and Isometric Force Simulations with Alternative Models 9

Supplementary Tables

Supplementary Table 1 Number of Observations and Molecules per Condition 10

Supplementary Table 2 Stroke Size Distributions Fitted Parameters at Saturating [ATP] 10

Supplementary Table 3 Global and Individual Fit Parameters for Durations at Saturating [ATP] 11

Supplementary Table 4 ATP independent dissociation rates from stopped flow experiments 11

Supplementary Table 5 Parameter Values for Simulations 12

Supplementary Notes

Supplementary Note 1 Deadtime Correction and Adjusted EC_{50} 13

Supplementary Note 2 Stopped-flow Experiments Show OM Does Not Speed Detachment from the Canonical AM-ADP or AM state. 13

Supplementary Note 3 Affinities and EC_{50} of OM for in vitro vs. Cardiomyocyte Experiments 14

Supplementary References 15

Supplementary Figures

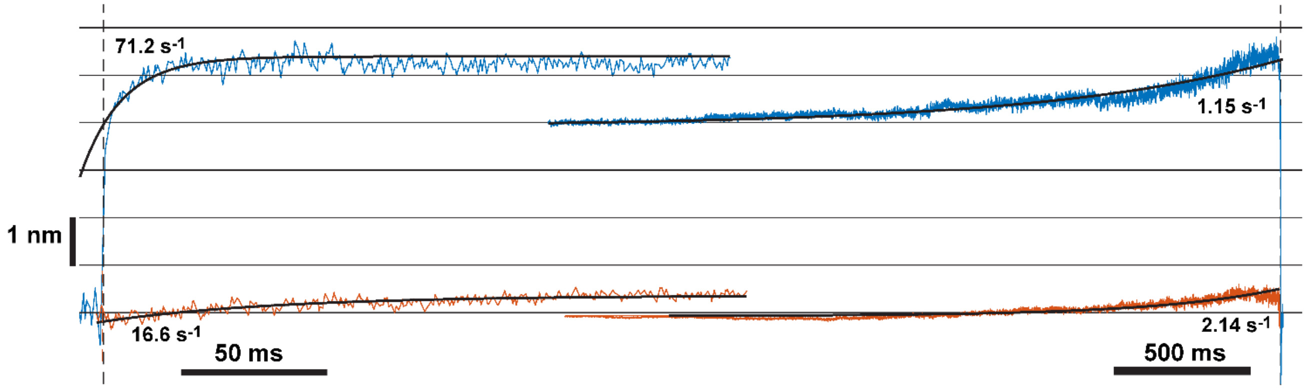

Supplementary Figure 1
Ensemble Averages of Myosin Working Stroke at 200 nM MgATP

The ensemble averages[1] with a 0.4 ms median filter applied in the absence of drug (blue) show a total step size of 5.7 nm with an initial displacement of 4.2 nm followed by a 1.5 nm sub step, consistent with previous results[2]. The rate constant of the forward ensemble average (left) is 71.2 s^{-1}, consistent with the rate of ADP release[3]. The rate constant of the reverse average (right), 1.15 s^{-1}, is close to the observed detachment rate and rate of ATP binding at 200 nM MgATP[3], which is to be expected at limiting ATP concentrations. In the presence of 10 μM OM (red), it is difficult to ensure proper alignment of the forward averages, so extracting information about the size and kinetics of the sub step is less reliable, but the total observed step is less than 0.5 nm, as is also the case at saturating MgATP. 600 events were averaged for the control case and 709 for the 10 μM OM case. Vertical dashed lines show the detected starts and ends of the events.

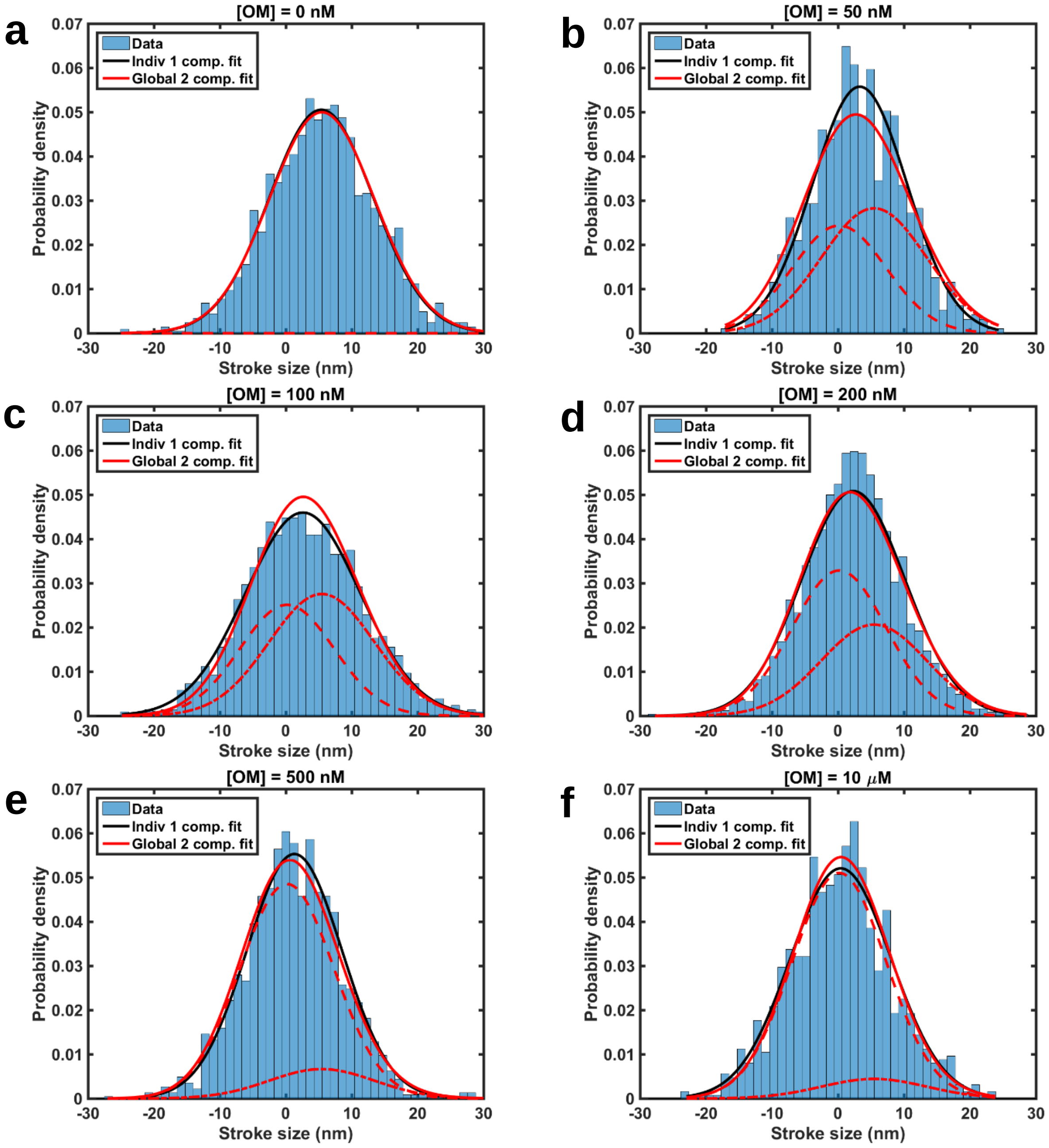

Supplementary Figure 2

Stroke Size Distributions at Saturating [ATP]

Stroke size distributions are shown for OM concentrations ranging from 0 to 10 µM (blue histograms). A single Gaussian distribution was fit to each dataset individually (black curves), and a 2 component Gaussian distribution was fit globally to all datasets, where the mean and standard deviations of the distributions were constrained to be shared among the datasets. The double Gaussian global fit is shown in solid red, and the large and small dashed curves show the contributions from the two separate components. One component had a mean of 0.18 nm (large dashed red) and the other of 5.46 nm (small dashed red). With increasing concentration of OM, the overall distribution shifts leftward toward zero and the contribution of the component with a stroke of 0.18 nm increases. Supplementary Figure 3a plots the fraction of events with the 5.46 nm stroke vs. OM concentration. Supplementary Table 1 gives the number of observations and Supplementary Table 3 lists all fitted parameters.

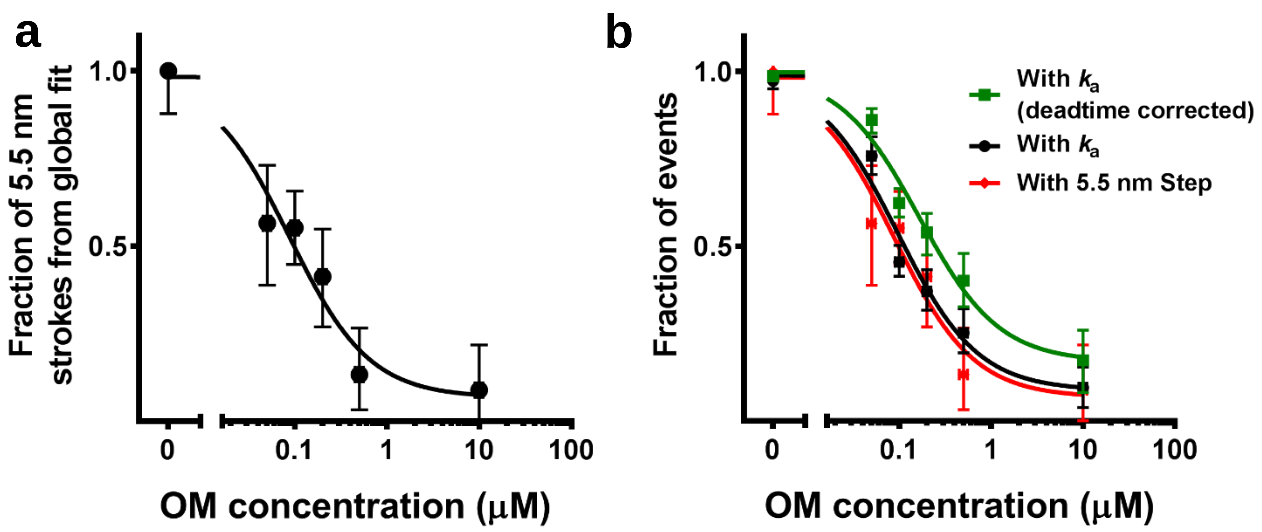

Supplementary Figure 3

Proportions of Events with 5.5 nm Stroke and Rate k_a as a Function of [OM]

(a) The fraction of interactions with a 5.46 nm step obtained from the global fitting of step size distributions is plotted against OM concentration. The dose dependent inhibition has an EC_{50} of 88 ± 31 nM (s.e.m.). **(b)** The data from panel a is replotted in red, alongside the fraction of events which dissociate at rate k_a (the rate observed in the absence of OM, black) which show an IC50 of 96 ± 27 nM. The close resemblance of these two curves supports the idea that OM acts in one way both to inhibit the step size and to prolong attachment duration when it is bound to myosin. The green plot shows the deadtime corrected fraction of events detaching at k_a, which takes into account that more events from this population are undetected due to the experimental deadtime compared to events that detach at the slower rate k_b (Supplementary Note 1). This correction shifts the EC_{50} to 164 ± 42 nM. Error bars show the 95% confidence intervals obtained via bootstrapping.

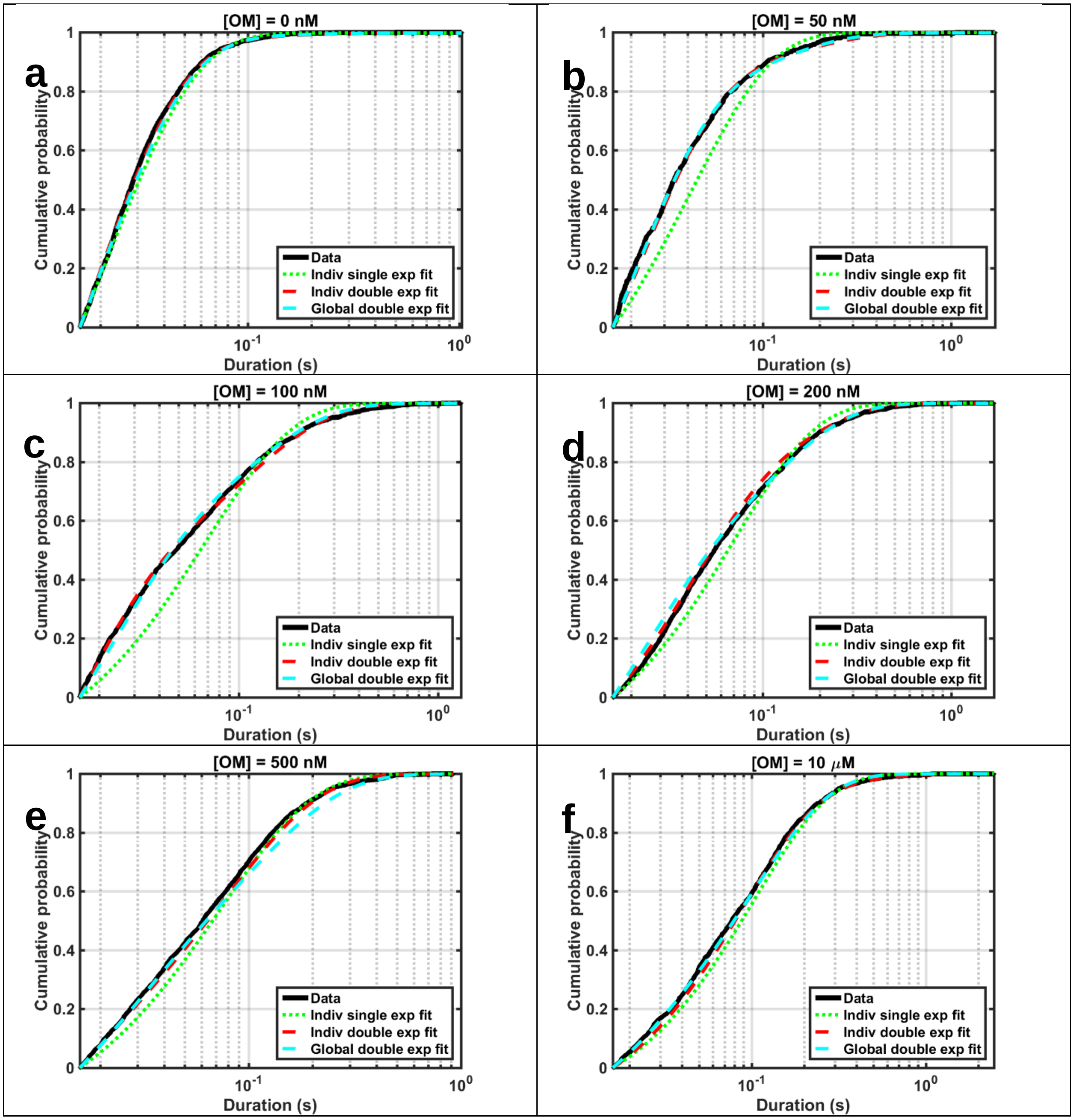

Supplementary Figure 4

Distributions and Fits of the Kinetics of Detachment at Saturating [ATP]

Cumulative density functions are shown for each OM concentration at saturating MgATP concentration on a semi-log scale (black). MLE Fits to a single exponential distribution only provide a good fit for the control data (a). Double exponential distributions fitted via MLE to each dataset individually (red) provide a much better fit. The global double exponential distribution (blue) forces all the of datasets to share the same two rates, but allows the proportion of events detaching at those rates to vary between datasets at different [OM]. These global fits provide nearly as good of a fit to all the datasets compared to the individual fits. The global fit requires 8 free parameters (2 rates and 6 amplitudes) to fit all datasets, whereas 18 free parameters are required for the separate double exponential fits. The number of observations are given in Supplementary Table 1 and p-values from the log-likelihood ratio test of MEMLET are given in Supplementary Table 3.

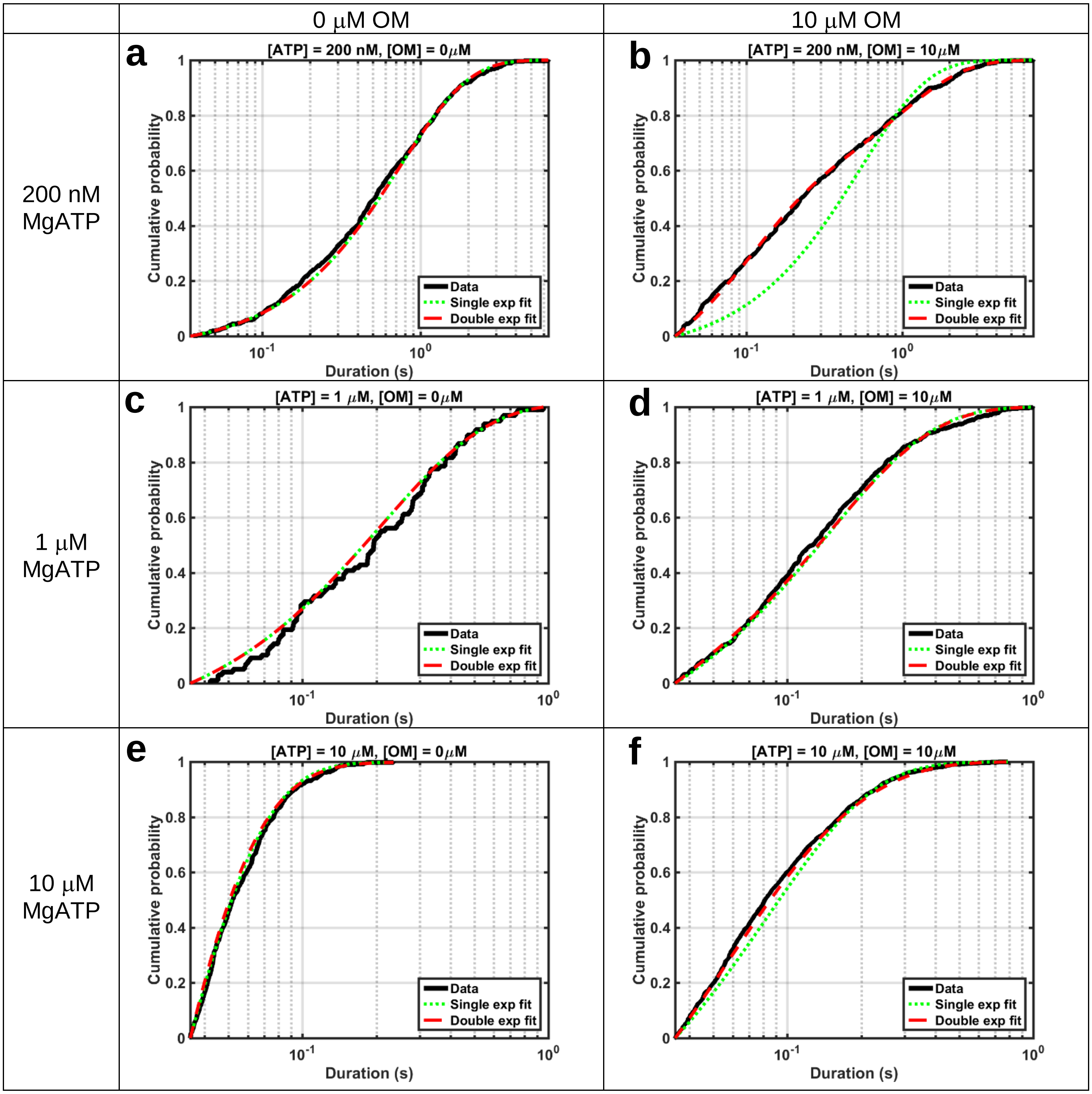

Supplementary Figure 5

Distributions and Fits of the Kinetics of Detachment at Sub-saturating [ATP]

Cumulative density distributions (black) of attachment durations at sub-saturating ATP concentrations for 0 and 10 μM OM (left and right columns respectively) on a semi-log axis. Note the x-axis is scaled differently for the 200 nM MgATP data (a and b). Single exponential distributions fit well to the data without OM (log-likelihood ratio test[4] for double exponential yielded p = 0.29, 0.44, and 0.43 for 200 nm, 1 μM, and 10 μM MgATP respectively), but double exponential distributions are required to describe the 10 μM OM data, especially at 200 nM and 10 μM MgATP (log-likelihood ratio test[4] for double exponential yielded p < 1e-15, = 0.023, and = 1.26e-12, for 200 nm, 1 μM, and 10 μM MgATP respectively). At 1 μM MgATP, the rate of ATP induced dissociation (5 per second) and OM-associated dissociation (10 per second) are so close, they become difficult to distinguish visually. The number of observations are given in Supplementary Table 1.

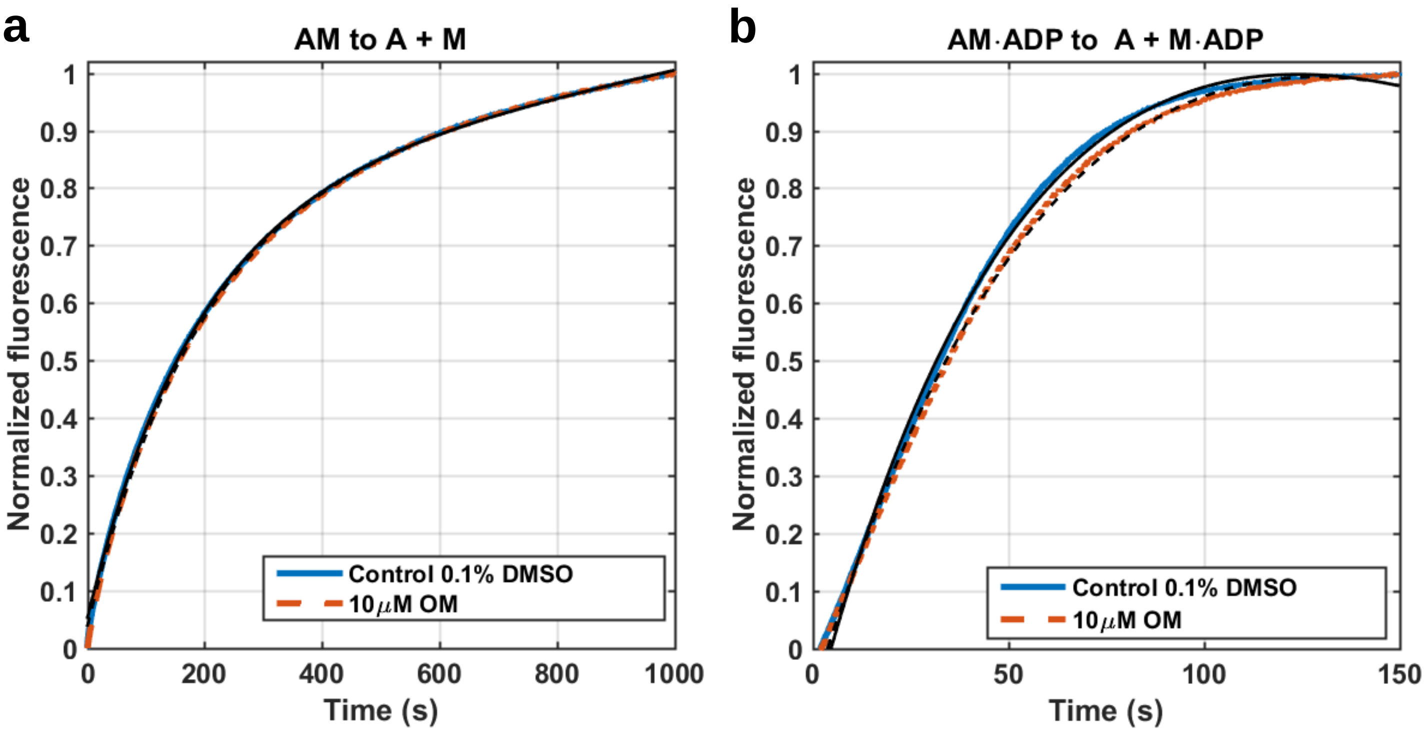

Supplementary Figure 6

Dissociation of Myosin from Actin from Nucleotide-free or ADP-bound States

(a) Normalized fluorescence transient increases of pyrene actin during the dissociation of rigor (nucleotide-free or apo) myosin from pyrene actin in the absence of OM (blue curve) and in the presence of 10 µM OM (red curve) as obtained from stopped-flow biochemical experiments. The data were fitted by a single exponential rise plus a constant slope (necessary likely due to the long duration of the experiment, black). The rate of detachment, was very slow, as expected (0.0054 s^{-1}), and was unchanged by OM. **(b)** We also observed the rate of dissociation of M-ADP from pyrene actin (blue, see Methods), which was faster than rigor, but still very slow (0.015 s^{-1}) and also nearly unchanged by the presence of OM (red, 0.012 s^{-1}). See Supplementary Table 4 for all rates.

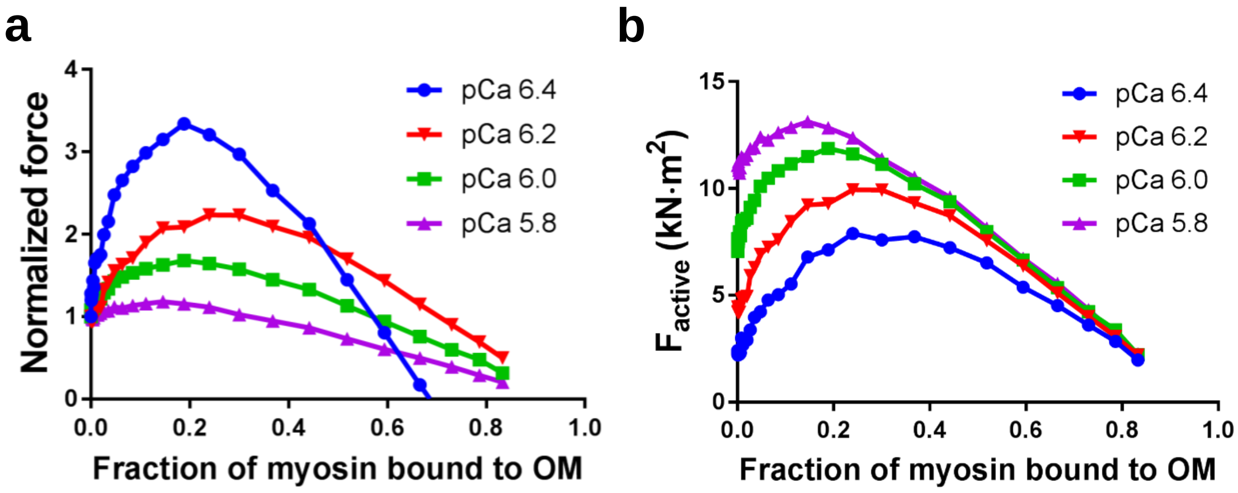

Supplementary Figure 7

Isometric Force as a Fraction of Myosin Bound to OM

The **(a)** normalized and **(b)** non-normalized isometric force developed at pCa 6.4-5.8 in simulations utilizing the SEPTA parameters from the table in Figure 5A plotted as a function of the fraction of myosin bound to OM. The bell-shaped response to force reaches a maximum between 15-35% of myosin bound to OM. In the simulations, pCa 6.4, 6.2, 6.0, and 5.8 correspond to 14%, 25%, 40%, and 63% of full activation respectively in the absence of OM.

a

	P_i rate (s^{-1})	$k_{Detach0}$ (s^{-1})	Stroke (nm)	d_{Bell} (nm)	Plot color
Control	17	89	5.5	1.3	
Malik Model	28	-	-	-	
SEPTA	28	10	0	0	
PTA only	28	10	-	0	
SE only	28	-	0	-	
Force Depend. ↑ 10x	28	-	-	13	
Pi Release Rate ↑ 10x	170	-	-	-	

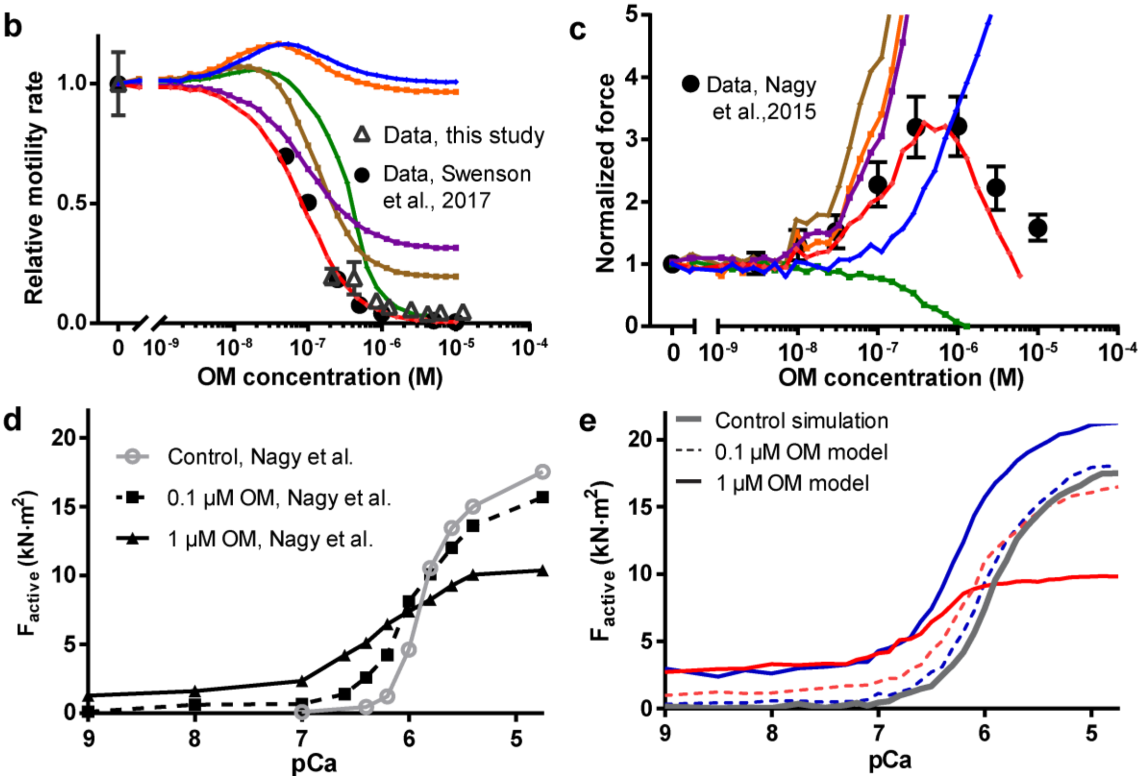

Supplementary Figure 8

Motility Rate and Isometric Force Simulations with Alternative Models

Simulations of OM's effect on gliding velocity and isometric force with additional models included for comparison to Figure 5 of the main text. **(a)** Summary of parameters used in models in panels (b)-(e). **(b)** Comparison of gliding filament velocity for the protein used in this study (open triangles) and from Swenson et al.[5] (closed circles) to simulated data from the various model parameter sets (colored lines as in (a)). Only the Stroke Eliminated, Prolonged Time of Attachment (SEPTA) model, with parameters from the single molecule measurements, fully accounts for the observed marked decrease of *in vitro* velocity as a function of OM concentration. Motility error bars are standard deviations of velocities from individual filaments (n of filaments= 92 - 6709). **(c)** Comparison of simulated, normalized, isometric forces at an intermediate calcium concentration (15% activation, lines colored as in (a)) and results from Nagy et. al (2015) who reported a bell-shaped curve of force development for permeabilized myocardial trabeculae as a function of OM (black circles). Only the SEPTA model (red) produces a similar biphasic shape. **(d)** Active force data reproduced from Nagy et. al. as a function of pCa (-log [Ca^{2+}]) at 0, 100 nM and 1 µM OM. **(e)** Simulated isometric force at 0 (grey line), 100 nM (dashed lines, colors as in (a)), and 1 µM OM (solid lines) for comparison with the experimental data in (d). Only the SEPTA model (red dashed and solid curves) recapitulates the measured leftward shift in the pCa-tension curve (calcium sensitization) and decreased force production at fully activating [Ca^{2+}].

Supplementary Tables

Supplementary Table 1
Number of Observations and Molecules per Condition

OM	ATP	n interactions	N molecules	N chambers	N days
**0 nM**	**200 nM**	600	3	2	2
**0 nM**	**1 µM**	619	4	3	2
**0 nM**	**10 uM**	1918	3	2	3
**0 nM**	**4 mM**	1676	8	8	8
**50 nM**	**4 mM**	779	4	4	4
**100 nM**	**4 mM**	1675	3	2	2
**200 nM**	**4 mM**	1993	3	3	2
**500 nM**	**4 mM**	1838	5	3	3
**10 µM**	**200 nM**	709	3	3	3
**10 µM**	**1 µM**	106	1	1	1
**10 µM**	**10 µM**	1588	3	2	2
**10 µM**	**4 mM**	1015	4	3	3

Supplementary Table 2
Stroke Size Distributions Fitted Parameters at Saturating [ATP]

OM	Global Two Component Fit					Estimated mean from global fit (nm)	Single Component Fit	
	Fraction with $mean_1$	$mean_1$ (nm)	$mean_2$ (nm)	$sigma_1$ (nm)	$sigma_2$ (nm)		mean (nm)	sigma (nm)
**0 nM**	0.000	0.183	5.463	7.132	7.986	5.463	5.395	7.897
**50 nM**	0.435					3.167	3.303	7.158
**100 nM**	0.449					3.094	2.597	8.680
**200 nM**	0.587					2.362	2.309	7.850
**500 nM**	0.867					0.885	1.300	7.217
**10 µM**	0.912					0.649	0.413	7.667

Supplementary Table 3
Global and Individual Fit Parameters for Durations at Saturating [ATP]

OM	Single Exp. Rate (s^{-1})	Individual Double Exp. Fit				Global Double Exponential Fit		
		k_a (s^{-1})	k_b (s^{-1})	Fraction with k_b	p-value for Indiv Dbl Exp	k_a (s^{-1})	k_b (s^{-1})	Fraction with k_b
**0 nM**	47.45	55.01	9.38	0.016*	<1e-15*	52.48	9.49	0.027
**50 nM**	24.08	47.11	7.60	0.106	<1e-15			0.242
**100 nM**	14.33	76.40	9.44	0.349	<1e-15			0.546
**200 nM**	14.01	29.43	8.04	0.334	<1e-15			0.629
**500 nM**	13.44	88.87	12.24	0.684	9.02e-10			0.748
**10 µM**	9.659	3.263	11.60	0.930	5.81e-10			0.905

*Note: Although this p-value is very small, due to the fraction of events with rate k_b being less than 2%, the single exponential fit is sufficient to account for >98% of the data

Supplementary Table 4
ATP independent dissociation rates from stopped flow experiments

	Rate Constant (s^{-1})	Uncertainty from fit (s^{-1})
**AM->M + DMSO**	0.0054	0.000009
**AM->M + 10 µM OM**	0.0052	0.000009
**AM·ADP->M·ADP + DMSO**	0.015	0.000105
**AM·ADP->M·ADP + 10 µM OM**	0.012	0.000093

Supplementary Table 5
Parameter Values for Simulations

Description	Parameter	Value	Source
Attachment Rate without OM (P_i rate)	f	17 s^{-1}	Liu et al.[3]
Attachment Rate with OM (P_i rate)	f_{OM}	Model dep*	Liu et al.[3]
Detachment Rate without OM	g	89 s^{-1}	This work
Detachment Rate with OM	g_{OM}	Model dep*	This work
Stroke size without OM	s	5.5 nm	This work
Stroke size with OM	s_{OM}	Model dep*	This work
Distance Parameter for Detachment without OM	d	1.3 nm	This work
Distance Parameter for Detachment with OM	d_{OM}	Model dep*	This work
Affinity of myosin for OM (motility)	OM K_D^{Motile}	0.1 μM^{-1}	This work
Affinity of myosin for OM (cardio myocyte)	OM $K_D^{Myocyte}$	1.2 μM^{-1}	Kampourakis et al.[6]
Stiffness of myosin	k_{myo}	0.5 pN/nm	Kaya et al.[7]
Coupling constant for thin filament activation **	$C=L/m_{space}$	11	Longyear et al.[8]
Scaling of rate f for off-state of thin filament	ε_{min}	0.0005	***
Scaling of rate f for on-state of thin filament	ε_{max}	0.5	Longyear et al.[8]
Affinity of Calcium for thin filament regulation	Ca K_D	2 μM^{-1}	Kobayashi et al.[9]

*See Supplementary Figure 8A for values

**This constant was used for simulations of motility assays and cellular-scale simulations in Longyear et. al., (2017) although it may not directly correspond to the cooperative unit in a native thin filament.

*** Adjusted to fit Nagy et al.[10] at 0 µM OM

Supplementary Notes

Supplementary Note 1
Deadtime Correction and Adjusted EC_{50}

The minimum detectable event in the experiments, derived from the window size used to calculate the covariance of the two beads, ranged from 16-30 ms depending on experimental conditions. As described previously[4], when two components of a kinetic process are observed with a deadtime, more events from the component with the faster process are undetected due to the deadtime as compared to those from the slower process. For example, in our data at 10 µM MgATP with no OM, we might expect the distribution of durations to be described the sum of two exponentials since the ADP release rate (~50 s^{-1}) is close the ATP binding rate (~30 s^{-1}). However, because of the 20 ms deadtime for detecting events from the bead covariance, the lag-phase expected from the ~50 s^{-1} ADP release rate is not observed, and the data are well fitted by a single exponential corresponding to the ATP binding rate.

In other cases, when fitting to a double exponential distribution is statistically justified (from the likelihood ratio test), this bias can lead to the fractional amplitude of the slow phase being over-estimated. The amplitudes of the phases presented in the main text (Figure 3C, 3D) show the observed amplitudes, without correction for the deadtime in order to simplify comparison with the step size data, for which no such correction can be made. When a correction for the deadtime is applied, the fraction of events occurring at rate k_a shifts, as shown in Supplementary Figure 3b (green curve), and the fitted EC_{50} increases from 96 ± 27 nM to 164 ± 42 nM. This may represent a more accurate estimate of the EC_{50} if the assumed model of two, single exponential components is correct. The proportion of steps with step size of 5.46 nm from the global fit (Supplementary Figure 3b, red) more closely resembles the observed amplitude of k_a (Supplementary Figure 3b, black), which is to be expected according to our model since myosin not bound to OM shows the 5.46 nm step and detaches more rapidly and thus the full step events are also more likely to be missed.

Supplementary Note 2
Stopped-flow Experiments Show OM Does Not Speed Detachment from the Canonical AM-ADP or AM state.

Since OM causes actomyosin dissociation at a rate independent of ATP concentration we sought to determine which biochemical state of myosin precedes this detachment. We performed stopped flow experiments using pyrene actin to measure the dissociation rate of actin-bound porcine cardiac myosin (S1) in the presence and absence of OM and with no nucleotide present (apo-myosin) or with MgADP. The detachment rates for both apo and ADP states were quite slow (<0.1 s^{-1}) in agreement with previously reported values for bovine cardiac myosin[11] and were unaffected by the presence of 10 µM OM (Supplementary Fig. 6; Supplementary Table 4). It is important to note that the ADP state in this study (AM·ADP) is that which is accessible via binding of ADP to the nucleotide-free (rigor) myosin molecule, and is not the AM'·ADP state that immediately follows phosphate release[12]. These data suggest that the OM-associated detachment we observe in the optical trapping experiments does not come from the canonical rigor or AM·ADP states.

Supplementary Note 3
Affinities and EC_{50} of OM for in vitro vs. Cardiomyocyte Experiments

The effective concentration yielding a 50% effect (EC_{50}) for OM varies by an order of magnitude between various reported experiments. Actin gliding assays using bovine or human β-cardiac myosin have EC_{50}'s ranging from 50 to 200 nM [3,5,13,14]. The EC_{50}'s measured in our optical trapping experiments fall within this range. However, inhibition of steady-state ATPase activity has been measured to have an EC_{50} of 500 nM for the same protein that exhibited an EC_{50} of 100 nM for gliding velocity[5]. In addition, the ATPase inhibition EC_{50} for permeabilized rat trabeculae has been reported as 1.2 μM[6]. The source of OM and preparation of stock solutions in this rat trabeculae study were the same as that used in our optical trapping and in vitro motility experiments. We used 1.2 μM as the affinity for our cardiomyocyte simulations (Fig 5 C, E) since this rat trabeculae preparation was similar to that used in the Nagy et. al. experiments[10].

The difference in the EC_{50} values may be explained either as isoform differences, differential affinity for nucleotide states of the myosin, or a combination of the two. Rat trabeculae contain a combination of α- and β-cardiac myosin, with 40-70% being α-cardiac myosin[15]. The affinity of OM for rat α- and β-cardiac myosin have not been measured, offering a possible explanation for the difference in observed affinities in the rat cardiomyocyte experiments versus the *in vitro* experiments using purified, human β-cardiac myosin. In addition, the trabeculae structure might contain hydrophobic pockets that could act as competitive binding sites for OM, which may cause a decrease in the observed EC_{50} of the drug as compared to the simplified in vitro systems which lack these hydrophobic pockets.

The affinity of OM for cardiac myosin has been directly measured via isothermal titration calorimetry for bovine cardiac myosin and ranges from 0.29 to 5.3 µM depending on the nucleotide state of myosin[16]. The tightest affinity was for an ADP·P_i analog, ADP·VO_4. Although the primary steady-state biochemical state for cardiac myosin in muscle is expected to be an ATP or ADP·P_i state, the relative population of the states, or the partition among other myosin states (e.g. the super-relaxed (SRX) state[17]) may affect the effective affinity of the drug. In addition, if OM only tightly binds to the ADP·P_i state, high actin concentrations may act as a type of competitive inhibitor to drug binding as myosin in the ADP·P_i state could either bind to OM or quickly bind to actin. Thus, the highly ordered sarcomere structure which positions actin close to myosin (creating a very large effective concentration), may serve to reduce the EC_{50} for the drug.

If the therapeutic plasma concentration of 100 - 600 nM is also present inside cardiomyocytes, it would result in approximately 50-80% of myosin bound to the drug based on our experimentally determined EC_{50} of 100 nM. This high percentage of drug-bound myosin would likely drastically reduce force production; however, it is not clear what is the internal concentration of the drug in patient cardiomyocytes. If the effective affinity of the drug was 1.2 μM, 200-600 nM OM would result in 15-30% of myosin being bound, consistent with increasing isometric force production at near physiological calcium levels due to thin filament cooperative activation (Supplementary Figure 7).

Supplementary References

1. Veigel, C. *et al.* The motor protein myosin-I produces its working stroke in two steps. *Nature* **398,** 530–533 (1999).
2. Greenberg, M. J., Shuman, H. & Ostap, E. M. Inherent Force-Dependent Properties of β-Cardiac Myosin Contribute to the Force-Velocity Relationship of Cardiac Muscle. *Biophys. J.* **107,** L41–L44 (2014).
3. Liu, Y., White, H. D., Belknap, B., Winkelmann, D. A. & Forgacs, E. Omecamtiv Mecarbil Modulates the Kinetic and Motile Properties of Porcine β-Cardiac Myosin. *Biochemistry (Mosc.)* **54,** 1963–1975 (2015).
4. Woody, M. S., Lewis, J. H., Greenberg, M. J., Goldman, Y. E. & Ostap, E. M. MEMLET: An Easy-to-Use Tool for Data Fitting and Model Comparison Using Maximum-Likelihood Estimation. *Biophys. J.* **111,** 273–282 (2016).
5. Swenson, A. M. *et al.* Omecamtiv Mecarbil Enhances the Duty Ratio of Human β-Cardiac Myosin Resulting in Increased Calcium Sensitivity and Slowed Force Development in Cardiac Muscle. *J. Biol. Chem.* **292,** 3768–3778 (2017).
6. Kampourakis Thomas, Zhang Xuemeng, Sun Yin-Biao & Irving Malcolm. Omecamtiv mercabil and blebbistatin modulate cardiac contractility by perturbing the regulatory state of the myosin filament. *J. Physiol.* **596,** 31–46 (2017).
7. Kaya, M. & Higuchi, H. Nonlinear Elasticity and an 8-nm Working Stroke of Single Myosin Molecules in Myofilaments. *Science* **329,** 686–689 (2010).
8. Longyear, T., Walcott, S. & Debold, E. P. The molecular basis of thin filament activation: from single molecule to muscle. *Sci. Rep.* **7,** 1822 (2017).
9. Kobayashi, T. & Solaro, R. J. Increased Ca2+ affinity of cardiac thin filaments reconstituted with cardiomyopathy-related mutant cardiac troponin I. *J. Biol. Chem.* **281,** 13471–13477 (2006).

10. Nagy, L. *et al.* The novel cardiac myosin activator omecamtiv mecarbil increases the calcium sensitivity of force production in isolated cardiomyocytes and skeletal muscle fibres of the rat. *Br. J. Pharmacol.* **172,** 4506–4518 (2015).

11. Siemankowski, R. F. & White, H. D. Kinetics of the interaction between actin, ADP, and cardiac myosin-S1. *J. Biol. Chem.* **259,** 5045–5053 (1984).

12. Sleep, J. A. & Hutton, R. L. Exchange between inorganic phosphate and adenosine 5'-triphosphate in the medium by actomyosin subfragment 1. *Biochemistry (Mosc.)* **19,** 1276–1283 (1980).

13. Aksel, T., Choe Yu, E., Sutton, S., Ruppel, K. M. & Spudich, J. A. Ensemble Force Changes that Result from Human Cardiac Myosin Mutations and a Small-Molecule Effector. *Cell Rep.* **11,** 910–920 (2015).

14. Winkelmann, D. A., Forgacs, E., Miller, M. T. & Stock, A. M. Structural basis for drug-induced allosteric changes to human β-cardiac myosin motor activity. *Nat. Commun.* **6,** 7974 (2015).

15. Lompré, A. M., Nadal-Ginard, B. & Mahdavi, V. Expression of the cardiac ventricular alpha- and beta-myosin heavy chain genes is developmentally and hormonally regulated. *J. Biol. Chem.* **259,** 6437–6446 (1984).

16. Planelles-Herrero, V. J., Hartman, J. J., Robert-Paganin, J., Malik, F. I. & Houdusse, A. Mechanistic and structural basis for activation of cardiac myosin force production by omecamtiv mecarbil. *Nat. Commun.* **8,** 190 (2017).

17. Hooijman, P., Stewart, M. A. & Cooke, R. A New State of Cardiac Myosin with Very Slow ATP Turnover: A Potential Cardioprotective Mechanism in the Heart. *Biophys. J.* **100,** 1969–1976 (2011).
